# Combined low-FOXP3+ and -CD3+tumor infiltrating lymphocytes: a signature of stage II MSS colorectal cancer at high-risk of recurrence

**DOI:** 10.1186/2051-1426-1-S1-P49

**Published:** 2013-11-07

**Authors:** Giuseppe Di Caro, Giuseppe Celesti, Fabio Grizzi, Paolo Bianchi, Gianluca Basso, Federica Marchesi, Alberto Mantovani, Alberto Malesci, Luigi Laghi

**Affiliations:** 1 Laboratory of Molecular Gastroenterology, Humanitas Clinical and Research Center, Milan, Italy; 2 Department of Immunology and Inflammation, Humanitas Clinical and Research Center, Milan, Italy; 3 Department of Gastroenterology, Humanitas Clinical and Research Center, Milan, Italy

## Background

Low densities of tumor infiltrating lymphocytes (TILs) predict colorectal cancer (CRC) post-surgical recurrence, the likelihood of progression also depending upon tumor stage and microsatellite (MS) status. We benchmarked FoxP3+TIL and CD3+TIL densities as predictor of postsurgical recurrence.

## Methods

FoxP3+ and CD3+TIL densities were measured as the percentage of immune-reactive area (IRA%) at the tumor invasion front, in a consecutive series of 413 patients (pts) undergone radical surgery for pT3-pT4, stage II (n=211) and III (n=202) CRC, characterized for MS status. Recursive partitioning, as well logistic univariate, interaction, and multivariate analysis were employed for the integrated examination of pathological and immune markers in predicting recurrence.

## Results

In a recursive classification tree, nodal status was the main prognostic factor, discriminating higher recurrence rate for stage III (70/202; 34.7%;) than for stage II (32/211, 15.2% P<.001) pts. Within stage II, 0.23%FoxP3+IRA cut-off divided recurrences in 8/127 (6.3%) pts with high (>0.23%) FoxP3+TILs, and in 24/84 (28.6%; P<.001) pts with low (≤0.23%) FoxP3+TILs. Within the latter group, CD3+IRA% further branched recurrences, that occurred in 7/39 (15.2%) pts with IRA>1.86%, but in 17/38 (44.7%; P=.003) pts with less IRA%. Differently, TIL densities did not predict recurrence in stage III. Interaction analysis revealed that the prognostic effects of FoxP3+and CD3+TIL densities were significantly (P<0.05) modified by nodal and MS-status status. ROC curve analysis in stage II pts with MSS CRC confirmed the cut-offs identified by recursive partitioning. At multivariate, low (<0.23%IRA) FoxP3+ and (<1.86% IRA) CD3+TILs were both independent recurrence predictors (O.R, 10.22 and 7.85, respectively; both P<.001), and concomitant low-FoxP3+ and -CD3+TILs conferred the highest recurrence risk (O.R. 12.64; P<.001). Among stage II pts, the low-FoxP3+ and -CD3+TIL signature identified 61% (17/28) of all MSS CRC recurrences, representing 71% (17/24) of post-surgical metastasis (O.R. 18.43; 95%C.I. 6.68-50.86), while none of 58 pts with high-FoxP3+ and -CD3+TIL experienced recurrences.

## Conclusions

FoxP3+ and CD3+TIL amount, plus tumor MS status efficiently stratify the risk of relapse of stage II CRCs, and may help in tailoring the post-surgical management of patients.

